# Association between Psychological Stress and Periodontitis: A Systematic Review

**DOI:** 10.1055/s-0039-1693507

**Published:** 2020-02-18

**Authors:** Micaele M. L. Castro, Railson de O. Ferreira, Nathalia C. F. Fagundes, Anna P. C. P. S. C. Almeida, Lucianne C. Maia, Rafael R. Lima

**Affiliations:** 1 Laboratory of Functional and Structural Biology, Institute of Biological Sciences, University Federal do Pará, Rua Augusto Corrêa 1, Guamá, Belém, PA, Brazil; 2 School of Dentistry, Faculty of Medicine and Dentistry, University of Alberta. 5528 Edmonton Clinic Health Academy, Edmonton, Canada; 3 Department of Pediatric Dentistry and Orthodontics, School of Dentistry, Universidade Federal do Rio de Janeiro, Rio de Janeiro, Brazil

**Keywords:** stress, psychological, periodontitis, periodontal disease, systematic review

## Abstract

This systematic review aims to investigate the association between psychological stress and periodontitis through analysis of cortisol levels and periodontal clinical parameters. This review was conducted according to the Preferred Reporting Items for Systematic Reviews and Meta-Analyses (PRISMA) guide and based on PECO (Participants, Exposure, Comparators, Outcomes) question and registered at PROSPERO under the code CRD42017076670. As eligibility criteria, observational studies performed in adult humans presenting periodontitis (P), which evaluated patients exposed (E) and nonexposed to psychological stress (C) and to verify the association between this type of stress and periodontitis (O) were included. The searches were performed until March 2018. The following databases were used: PubMed, Scopus, Web of Science, The Cochrane Library, LILACS, OpenGrey, and Google Scholar. After searches, the duplicate results were removed. The remaining citations were selected according to eligibility criteria in two phases. In the first phase, the title/abstract was evaluated. In the second phase, the articles were chosen previously were assessed by full text. After selection, the studies were submitted to data extraction and risk of bias evaluation by Fowkes and Fulton. A total of 1,386 citations were retrieved. After duplicates removal and selection process, three articles were selected by full text. Among them, two articles reported a positive association between psychological stress and periodontitis. All articles were classified as low risk of bias. Even though two articles highlighted an association between psychological stress and the presence of a possible modulatory pattern of cortisol levels in clinical parameters of periodontitis, more studies are necessary to elucidate this question.

## Introduction

The periodontitis is a chronical inflammation caused by bacteria, mostly gram negative. 1 The interaction of systemic conditions and oral microbiota modulates the severity of this disease. 2 3 As a consequence of this process, damage to periodontal tissue, especially on the alveolar bone and periodontal ligament, can be observed. 4 5

The beginning and the progression of periodontitis, as well as other chronical diseases, are associated with many risk factors, such as diabetes, smoking, age, and genetic predisposition. 6 7 Psychological factors, such as anxiety and depression, are associated with changes in immune response, which can increase periodontitis susceptibility. 8 9 10 11

The stress also presented a role of modulation in immune response 12 due to their reduction in defense capacity. As a consequence of this mechanism, the organism turns more susceptible to develop psychosomatic and inflammatory diseases. 13 14 This modulation may favor the beginning or progression of periodontal disease 15 16 since stressors can aggravate damage to the supporting and protect tissues of the tooth. 17

This systematic review sought to investigate the clinical evidence of the association between chronical psychological stress and periodontitis in humans.

## Methods

### Protocol and Registry

This systematic review was registered at PROSPERO under the code CRD42017076670. This database is from University of York and is responsible for recording and disseminating systematic reviews. This systematic review was performed according to Preferred Reporting Items for Systematic Reviews and Meta-Analyses (PRISMA) 18 guidelines and the Cochrane's protocol ( Supplementary Table S1 , online only). 19

### Selection Criteria

In this review, we aimed to answer the following question: “Is the psychological stress is associated with periodontitis?.” The eligibility criteria were defined according to PECO strategy. This acronym represents the patient (P), exposition (E), comparison (C), and outcome (O) characteristics of the eligible question. Only observational studies, with an adult human population (P), which evaluate exposed (E) and nonexposed patients to psychological stress (C), assess the association between stress and periodontitis (O) were included in this review.

Case reports, reviews, descriptive studies, opinion articles, technical articles, animal studies, and in vitro studies were excluded.

### Search Strategy

The searches were performed on the following electronic databases: PubMed, Scopus, Web of Science, Lilacs, and Cochrane Library. The Google Scholar and OpenGrey were used as gray literature sources. No restriction of year or language of publication was applied. The search strategy was composed of MeSH and free terms and was adapted according to each database ( Supplementary Table S2 , online only).

The searches were performed until March 2018. Additionally, an alert was created in each database to retrieve new studies according to eligibility criteria. After searches, the citations found in each database were exported to a reference manager software (EndNote, X7 version, Thomson Reuters, Philadelphia, United States). The articles presented in more than one database were considered only once.

### Process of Selecting Studies

After the importation of citations, the duplicate results were removed. The selection process was divided into two phases, according to eligibility criteria. In the first phase, all citations were evaluated by two reviewers (MMLC and ROF) and checked by a third reviewer in case of disagreement (RRL), regarding title and abstract. In phase II, the articles selected on phase I were evaluated by full-text, following the same criteria and method described on phase I. Additionally, the references of the chosen articles on phase II were checked for further studies.

### Quality Assessment and Risk of Bias

The Fowkes and Fulton's checklist 20 was used in this systematic review to evaluate the quality and risk of bias of the included studies. In this checklist, the quality of the articles was assessed by seven central domains: “Study design appropriate to the objective?”; “Study sample representative?”; “Control group acceptable?”; “Quality of measurements and outcomes?”; “Completeness?”; “Distorting influences?”

For each question, it was attributed a 0 (no problem), + (minor problem) or ++ (major problem). The criteria for this evaluation were standardized by evaluators and adapted from Fowkes and Fulton 20 and Almeida et al 21 ( Table 1 ).

**Table 1:** Domains and risk of bias considered in risk of bias evaluation according to Fowkes and Fulton

Guidelines	Checklist	Description
**Study design appropriate to objectives?**	Objective common design	The type of study was marked in the appropriate type of study. If the type of study was appropriate according to the study design, it was labeled as “0,” and as “++” if it was not appropriate
	Prevalence cross-sectional	
	Prognosis cohort	
	Treatment controlled trial	
	Cause cohort, case–control, cross-sectional	
**Study sample representative?**	Source of sample	The domain was considered “0” in cases of detailed origin, “+” to a specified origin of only one group and “++” in cases of absence of specification of the source of the groups
	Sampling method	The item was assigned “0” for a full description of sampling method, “+” for poor or no explanation of sample method, with no problem in matching between groups, and “++” for poor or no description of sample method, interfering in the matching of the groups
	Sample size	A minor problem “+” was considered when the sample was not representative or did not report a sample calculation. To a major problem, “++” was considered when no sample calculation was provided, and the number of participants was less than 50 participants, “0” was considered in the absence of the above factors
	Entry criteria/exclusion	A minor problem “+” was attributed when the control and case group reported current use of antibiotics or anti-inflammatories, diabetes, smoking or pregnancy. In the case of presence of more than two previously mentioned items, it was considered as a major problem “++”
	Nonrespondents	The “0” was attributed when there was no refusal to participate in the study, “+” was assigned when there was the refusal, but did not compromise the sample, and “++” when there were refusal and impairment of the sample size
**Control group acceptable?**	Definition of controls	It was attributed “0” when all characteristics of the control group were described, “+” when any information was pendent as the origin of the control group, the selection criterions and a different origin between case and control groups and “++” when two or more items described in previously items
	Source of controls	It was considered “0” when the control group was referred, “+” when the origin of groups was different, but with reasons and “++” when the groups presented different origins without reasons
	Matching/randomization	In this item, “0” was assigned to cases of randomized/matched groups, “+” to cases of no description of randomization, but with a matching of groups and “++” to no explanation of randomization or matching
	Comparable characteristics	It was attributed “0” to matched groups or not matched by the impossibility of being subsequently adjusted and “++” the presence of unpaired variables that were not paired or adjusted
**Quality of measurements and outcomes?**	Validity	It was considered “0” when the evaluation method applied is appropriate; “+” when using a single method, but with appropriate sensitivity with good specificity; “++” when using a single method, without an adequate specificity or good sensitivity
	Reproducibility	It was considered “0” whether the evaluation methods were well described; “+” when a lack description of any step of the method was presented, for example, the identification of the patients of the groups studied in laboratory samples, evaluations at different times or application of various methods between groups of individual pathology; “++” when two or more of the previous items are present
	Blindness	The condition of the study participants was considered to be “Blind,” in this case being assigned the signal “0,” in cases of “not blind” the signal “++” was attributed
	Quality control	It was considered a problem when the examiner was not qualified; a partial periodontal exam was performed [not in all teeth or not in all the six periodontal sites/teeth], the measurement of periodontitis was only radiographic or the absence of the number of evaluated teeth sites. A minor problem “+” was considered when two of these characteristics were present, and a major problem “++” if more than two of these characteristics were present
**Completeness**	Compliance	It was assigned “0” for a sample size that remains the same from the beginning to the end or decreases without compromising the power of the test; “+” for differences in sample size at the end of the study, compromising the power of the test, but with reasons and adjusts; “++” for difference in sample size at the end of the study, compromising the power of the test, without reasons
	Dropouts	The “0” was scored when there is no loss during the study, “+” when there is a withdrawal that involves the inclusion criteria, such as age, sex, “++” when there is withdrawal and it compromises more than one criterion
	Deaths	This item was scored as Not Applicable “NA,” due to the type of PECO strategy
	Missing data	In this item, “0” was assigned to cases of randomized/matched groups, “+” to cases of no description of randomization, but with a matching of groups and “++” to no description of randomization or matching
**Distorting influences?**	Extraneous treatments	In this item, “0” was considered when there were no external influences; “+” when there are external influences, but that does not interfere in the results; “++” when there are external influences and interferes with the results
	Contamination	This item was scored as Not Applicable “NA,” due to the type of PECO strategy
	Changes over time	In this item, “0” was attributed to data collected in the same period; “+” to data obtained from the control group and the study group at different times that may cause distortions; “++” when the previous item was associated with data from studies already published
	Confounding factors	A problem was assigned when the data analysis involved enrollment of persons < 5 years. Menopausal woman, smokers, diabetics and obese. A minor problem “+” was assigned when 1 or 2 of these characteristics were present and a major problem “++” if there were 3 or more
	Distortion reduced by analysis	It was considered “0” when it cites the adjustments of the covariates that present distortions; “+” when the article report adjustment, but does not say the criteria; “++” when distortion was identified, without adjustment
**Summary questions**	Bias: Are the results erroneously biased in a certain direction?	YES or “NO” answers were assigned to each question. If the answer is NO to the three questions, the article is considered reliable, with low risk of bias
	Confounding: Are there any serious confusing or other distorting influences?	
	Chance: Is it likely that the results occurred by chance?	

The risk of Bias was evaluated following three summary questions presented at the end of the checklist: “Bias: Are the results erroneously biased in a certain direction?,” “Confounding: Are there any serious confusing or other distorting influences?” and “Chance: Is it likely that the results occurred by chance?” In each question, a “Yes” or “No” was attributed to an answer. In case of a “No” answered in all questions, the study was considered as a low risk of Bias.

### Data Extraction

The data regarding the country, year, study design, sample characteristics (sample source and size), age, periodontitis evaluation, stress evaluation (cortisol levels measurement), results, and statistical analysis were extracted from all articles included after the selection process. This process was performed by two reviewers (MMLC and ROF) and checked by a third reviewer in case of disagreement (RRL).

## Results

### Studies Included

**Fig. 1:**
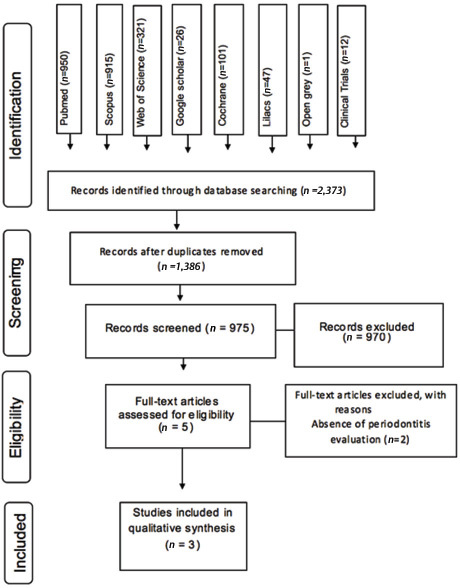
Flow diagram of literature search according to Preferred Reporting Items for Systematic Reviews and Meta-Analyses (PRISMA) statement.

A total of 2,373 articles were retrieved after searches and 1,386 remained after the exclusion of duplicates. From these, five articles were selected on phase I. 22 23 24 25 26 Among them, two articles were excluded due to the absence of periodontitis evaluation 23 26 ( Fig. 1 ).

### Description of the Study Characteristics

As a result, three articles were included in this review. According to study design, two were cross-sectional studies, 24 25 and the other one was a case–control 22 ( Table 2 ).

**Table 2:** Summary of characteristics of the included studies

Author, year, country; design	Sample		Age				Results
			Mean (±SD), in years	Methods of evaluation			
	Source	Size		Periodontitis	Cortisol levels	Statistical analysis	
**Ishisaka et al 2008** 25 **; Japan; cross-sectional**	Fukuoka, Japan	467	62.6±2.89	CAL, BOP, and PD.	Radioimmunoassay, seric levels.	Kruskal–Wallis	An association between cortisol and CAL levels was detected ( *p* = 0.11), with higher levels of cortisol in sites with elevated CAL
		Control: 107					
		Stress: 187					
**Hilgert et al 2006** 24 **; Brazil; cross-sectional**	Porto Alegre, Brazil	235	Control: 61.4±7.2	CAL, BOP and PD.	Radioimmunoassay, salivary levels.	Multivariate logistic regression	Cortisol levels were positively associated with a CAL ≥ 4 mm (OR = 5.1, 95% CI [1.2, 20.7]) *;* 30% of sites with CAL ≥ 5 mm (OR = 6.9, 95% CI [1.7, 27.1]); and 26% of sites with PD ≥ 4 mm (OR = 10.7, 95% CI [1.9, 54.1]) after adjusting for confounding variables
		Control: 212	Stress: 61.6±8.3				
		Stress: 23					
**Bakri et al, 2013** 22 **; United Kingdom; case–control**	Sheffield, United Kingdom	45	Control: 49.8±9.7	CAL, BOP, PD, tooth mobility, Gingival recession	ELISA (salivary levels)	Student's t-test	No difference between means of CAL and PD IN stressed and nonstressed patients was detected
		Control:16	Stress: 44.6±10.4				
		Stress: 29					
Abbreviations: BOP, bleeding on probing; CAL, clinical attachment loss; CI, confidence interval; OR, odds ratio; PD, probing depth; SD, standard deviation.							

Two articles reported an association between psychological stress and periodontitis 24 25 ^,^ indicating an increase on clinical attachment loss (CAL), probing depth (PB), and bleeding on probing. One study 22 did not report an association between the evaluated conditions, indicating similar levels of PD and CAL in stressed and nonstressed patients.

Regarding the stress evaluation, only studies that included the cortisol levels measurement were added, due to the increase in these levels caused by psychological stress. 13 Two articles measured salivary cortisol levels 22 24 and Ishisaka et al 2008 evaluated serum cortisol. These levels were measured by radioimmunoassay 24 25 and enzyme-linked immunosorbent assay 22 on the included studies.

### Quality Assessment and Risk of Bias

During our analysis, we considered that some factors to reduce the risk of bias could favor the applicability of this review and promote conclusions of the association between stress and periodontitis. Among them, the inclusion and exclusion criteria approved groups that presented similar characteristics differing only in the presence/absence of stress criterion. Thus, the variables stress and periodontitis were the primary variables related to the outcome.

All studies were classified with low risk of bias. The main problems observed among articles were related to sampling method, sample size, randomization/matching, and confounding factors ( Table 3 ).

**Table 3:** Quality assessment of studies included, according to Fowkes and Fulton, 1991

Guideline	Checklist	Hilgert et al 2006 24	Ishisaka et al 2008 25	Bakri et al 2013 22
**Study design appropriate to objectives?**	Objective common design	–	–	–
	Prevalence cross-sectional	–	–	–
	Prognosis cohort	–	–	–
	Treatment controlled trial	–	–	–
	Cause cohort, case–control, cross-sectional	0	0	0
**Study sample representative?**	Source of sample	0	0	0
	Sampling method	++	++	++
	Sample size	+	+	0
	Entry criteria/exclusion	0	0	0
	Nonrespondents	+	0	0
**Control group acceptable?**	Definition of controls	0	0	0
	Source of controls	0	0	0
	Matching/randomization	+	+	+
	Comparable characteristics	0	0	0
**Quality of measurements and outcomes?**	Validity	0	0	+
	Reproducibility	0	0	0
	Blindness	0	0	0
	Quality control	0	0	0
**Completeness**	Compliance	0	0	0
	Dropouts	0	0	0
	Deaths	NA	NA	NA
	Missing data	0	0	0
**Distorting influences?**	Extraneous treatments	0	0	0
	Contamination	NA	NA	NA
	Changes over time	0	0	0
	Confounding factors	+	+	0
	Distortion reduced by analysis	0	0	0
**Summary questions**	Bias: Are the results erroneously biased in a certain direction?	No	No	No
	Confounding: Are there any serious confusing or other distorting influences?	No	No	No
	Chance: Is it likely that the results occurred by chance?	No	No	No
0 = no problem; + = minor problem; ++ = major problem; NA = not applicable.				

All studies presented a major problem on sampling method due to the absence of a random sample. 22 24 25 Regarding sample size, a minor problem was reported in two studies 24 25 due to the lack of sample size calculation.

In the domain “Control group acceptable?,” all articles presented a minor problem due to the absence of description of randomization/matching process.

The selected articles 22 24 25 presented valid methods periodontitis and stress evaluation, such as the American Academy of Periodontology classification 27 and the assessment of salivary and blood cortisol, respectively. However, a minor problem was attributed to validity question on Bakri et al 2013 22 part of the domain: “Quality of measurements and outcomes?” This problem was observed due to the absence of calibration among evaluators.

Besides the standardized methods, the presence of blinded evaluators and the use of statistical methods to reduce the confounding factors may reduce the risk of Bias of the included studies.

## Discussion

In this systematic review, three articles respected the eligibility criteria and were included. Among them, two studies reported an association between psychological stress and periodontitis. 24 25

Systematic reviews judiciously gather studies with the purpose of synthesizing the clinical situations through strategies that allow the critical evaluation of these studies. This research method helps to summarize clinical questions and the clinical decisions of medical professionals. 28

This systematic review aimed to evaluate the association between psychological stress and periodontitis. Association could mean (1) that periodontitis is caused by stress, (2) that stress is caused by periodontitis, and finally (3) that both phenomena are just correlated because they a driven by other factors. In this case, specially, our PECO strategy helps our study to find studies that research the influence of the psychological stress on periodontitis. Other important difference is that each included study our systematic review evaluates stress marker and clinical parameters, excluding articles that subjectively analyze from both conditions.

This systematic review aimed to evaluate the association between psychological stress and periodontitis. Among the three included studies, 22 24 25 two reported that higher levels of cortisol were associated with a worsening of periodontitis. 24 25 The cortisol, glucocorticoid stress biomarker, is responsible for maintaining the homeostasis of the organism. 29 However, an exacerbated production of this substance can result in nocive effects, such as deregulation of the immune response and changes in inflammatory modulation. Hypothalamic–pituitary–adrenal axis produces the cortisol production as a result of stress, which triggers this response on the central nervous system. 13 16 30

In a long term, the cortisol can reduce the ability of the immune system by inhibiting immunoglobulins A and G, altering the T-helper and T-suppressor balance, and causing modifications of Natural Killer cells. 31 32 33 This combination of changes in inflammatory responses and depression of the immune system caused by elevated levels of cortisol in the body lead to the recognition of psychological stress as a potential factor for the pathogenesis of chronic diseases such as periodontitis. 32 34

The literature highlights other pathophysiological factors that explain the principles of association among stress, high levels of cortisol and periodontitis. Studies in humans point out that psychological stress presents a relationship with periodontitis, elucidating elevated levels of IL-1β in patients who presented both conditions. 35 36 37 The consequence of the imbalance of this cytokine deregulates the host response and also the resistance to pathogens, consequently aggravating damages in chronic lesions such as periodontitis. 38

In animal model studies, greater periodontal destruction was demonstrated in stressed rats with an increase in pro-absorption factor (RANKL). 39 The receptor activator of nuclear factor kappa-B ligand (RANKL) is a chemotactic factor responsible for forming and activating osteoclasts 39 from macrophages precursor cells. Also, the proliferation of anaerobic bacteria and subsequent damage to the periodontium are favored with the increase in oxygen metabolism in the periodontal tissues. 40 41

The methods of measurement of salivary and serum cortisol are used for the diagnostic categorization of chronic stress. Both methods are characterized as viable to obtain parameters that configure the stress. The serum cortisol evaluated by chemiluminescence and immunoassay techniques are the primary methods of choice in stress analysis. However, such techniques present biases related to the low specificity of the analysis antibodies and their considerable affinity for other steroidal hormones. 42

Thus, the salivary cortisol measurement method has become more popular due to the diffusion of cortisol to saliva independently of the salivary flow, the ease of sample collection, and the better differentiation of analysis antibody bindings to steroid hormones. 43 44 Only one study evaluated serum cortisol 25 in this study all included patients are systemically healthy. Hilgert et al 24 and Bakri et al 22 use salivary cortisol measurement.

Besides blood and saliva, the increased cortisol levels in the gingival crevicular fluid were also associated with the severity of periodontitis. 43 44 45 This is an aggravating factor in the disease in the absence of adequate treatment for the periodontal tissue in stressed patients. 5 46

As for the analysis described in the articles selected for periodontitis, the World Health Organization advises that periodontal treatment should be recommended in teeth that have periodontal pockets above 3 mm. For this, the PB examination should be performed. PB and the Community Periodontal Index of Treatment Needs are safe parameters for complete analysis of the presence, extent, and severity of periodontitis. 47 In this review, all studies conducted PB and CAL for the diagnosis of periodontitis. 22 24 25

In this context, PB ≥4 mm and CAL ≥5 mm are references adopted for moderate-to-severe periodontitis 47 48 and used as a diagnostic method for periodontitis. Both PB values, 5 and 6 mm, are characteristic of interventions that involve surgical procedures to reduce the clinical aspect of insertion loss. 47 48 Therefore, factors such as stress may be related to this progression of severity in such patients.

To qualify the methods used in the studies, the Fowkes and Fulton 20 checklist, adapted from Almeida et al, 21 was adopted. This checklist assessed whether the methods applied in the observational studies are sufficient to produce coherent and useful information.

Bakri et al, 22 presented more considerable methodological problems than other articles. 24 25 The methods used to analyze the association between the studied conditions need a more reliable evaluation of periodontitis. Even though this study has used validated indexes, the absence of calibration of the evaluators increases discrepancies in the results obtained. Besides minor problems presented in the included studies from this review, a total of 747 articles were evaluated in three studies; it has been considered a good sample to reunite in a systematic review. 49

It is remarkable that more studies have to be performed to elucidate this association. These studies have to evaluate not only methodologies measurables that evaluate cortisol levels and periodontal clinical parameters but also that follow-up patients for months or years to evaluate the influence of psychological stress on the initiation and progression of periodontitis. Interventional studies can be done too, to answer the dentistry and psychological therapeutics influence during periodontitis.

The selected articles suggest that stress is a relevant psychosocial factor and may be part of the various agents responsible for the multifactorial of the etiology of periodontitis 13 16 17 30 50 These two comorbidities have a plausible physiopathological basis, and the association between them indicates that the maintenance of periodontal health is essential in patients with psychological stress. Also, if these patients undergo periodontal therapy, the response to treatment may be unfavorable. 46 51

## Conclusion

Although two articles highlight psychological stress as a modulator of alterations in periodontitis, more research is needed on this relationship using more sensitive methodological tools. It is important to emphasize the importance of new research that relates high levels of cortisol in the body to the level of alveolar bone loss, as well as longitudinal and interventional studies that assess whether stress therapies can contribute to the improvement of periodontal health in patients undergoing treatment of periodontitis.
