# Supplementary material for: Association between Psychological Stress and Periodontitis: A Systematic Review
Source: Eur J Dent. 2020 Feb 18;14(1):171–9. doi: 10.1055/s-0039-1693507 (PMC7069755; doi:10.1055/s-0039-1693507)
Supplement: Supplementary file 2 — Supplementary Material [file 10-1055-s-0039-1693507_00016_s2.pdf]

Supplementary Table S2 Terms used on database search

Database	Search Format
PUBMED	(((((((((((Humans[MeSH Terms]) OR Humans[Title/Abstract]) OR Man, modern[Title/Abstract]) OR Modern Man[Title/Abstract]) OR Man Taxonomy[Title/Abstract]) OR Homo sapiens[Title/Abstract]) OR Human[Title/Abstract]) OR adults[MeSH Terms]) OR adults[Title/Abstract]) OR adult[Title/Abstract])) AND ((((((((((((((((((((((((((Hydrocortisone[MeSH Terms]) OR Hydrocortisone[Title/Abstract]) OR Cortisol, dehydroepiandrosterone[Title/ Abstract]) OR Cortisol[Title/Abstract]) OR Hydrocortisone, (9 beta,10 alpha,11 alpha)-Isomer[Title/Abstract]) OR Hydrocortisone, (11 alpha)-Isomer[Title/Abstract]) OR 11-Epicortisol[Title/Abstract]) OR 11 Epicortisol[Title/Abstract]) OR Epicortisol[Title/Abstract]) OR Cortifair[Title/Abstract]) OR Cortril[Title/Abstract]) OR Stress, Psychological[MeSH Terms]) OR Stress, Psychological[Title/Abstract]) OR Psychological Stresses[Title/Abstract]) OR Stresses, Psychological[Title/Abstract]) OR Life Stress[Title/Abstract]) OR Life Stresses[Title/Abstract]) OR Stress, Life[Title/Abstract]) OR Stresses, Life[Title/Abstract]) OR Stress, Psychologic[Title/Abstract]) OR Psychologic Stress[Title/Abstract]) OR Psychological Stress[Title/Abstract]) OR Mental Suffering[Title/Abstract]) OR Suffering, Mental[Title/Abstract]) OR Suffering[Title/Abstract]) OR Anguish[Title/Abstract]) OR Emotional Stress[Title/Abstract]) OR Stress, Emotional[Title/Abstract])) AND ((((((((((((((((((((((((((((((((((((((((((((((Periodontitis[MeSH Terms]) OR Periodontitis[Title/Abstract]) OR Pericementitis[Title/Abstract]) OR Pericementitides[Title/ Abstract]) OR Periodontal Diseases[MeSH Terms]) OR Periodontal Diseases[Title/Abstract]) OR Disease, Periodontal[Title/Abstract]) OR Diseases, Periodontal[Title/Abstract]) OR Periodontal Disease[Title/Abstract]) OR Parodontosis[Title/Abstract]) OR Parodontoses[Title/Abstract]) OR Pyorrhea Alveolaris[Title/Abstract]) OR Periodontal Atrophy[MeSH Terms]) OR Periodontal Atrophy[Title/Abstract]) OR Periodontal Atrophies[Title/Abstract]) OR Atrophy of Periodontium[Title/Abstract]) OR Periodontium Atrophies[Title/Abstract]) OR Periodontium Atrophy[Title/Abstract]) OR Gingivo-Osseous Atrophy[Title/Abstract]) OR Gingivo Osseous Atrophy[Title/Abstract]) OR Gingivo-Osseous Atrophies[Title/Abstract]) OR Alveolar Bone Loss[MeSH Terms]) OR Alveolar Bone Loss[Title/Abstract]) OR Alveolar Bone Losses[Title/Abstract]) OR Alveolar Process Atrophy[Title/Abstract]) OR Alveolar Process Atrophies[Title/Abstract]) OR Alveolar Resorption[Title/Abstract]) OR Alveolar Resorptions[Title/Abstract]) OR Resorption, Alveolar[Title/Abstract]) OR Resorptions, Alveolar[Title/Abstract]) OR Bone Loss, Periodontal[Title/Abstract]) OR Bone Losses, Periodontal[Title/Abstract]) OR Periodontal Bone Losses[Title/Abstract]) OR Periodontal Bone Loss[Title/Abstract]) OR Periodontal Resorption[Title/Abstract]) OR Periodontal Resorptions[Title/Abstract]) OR Resorption, Periodontal[Title/Abstract]) OR Alveolar Bone Atrophy[Title/Abstract]) OR Alveolar Bone Atrophies[Title/Abstract]) OR Bone Atrophies, Alveolar[Title/Abstract]) OR Bone Atrophy, Alveolar[Title/Abstract]) OR Bone Loss, Alveolar[Title/Abstract]) OR Chronic Periodontitis[MeSH Terms]) OR Chronic Periodontitis[Title/Abstract]) OR Chronic Periodontitides[Title/ Abstract]) OR Periodontitides, Chronic[Title/Abstract]) OR Periodontitis, Chronic[Title/Abstract]) OR Adult Periodontitis[Title/Abstract]) OR Adult Periodontitides[Title/ Abstract]) OR Periodontitides, Adult[Title/Abstract]) OR Periodontitis, Adult[Title/Abstract])

(*continued*)

Supplementary Table S2 (*continued*)

Database	Search Format
SCOPUS	((TITLE-ABS-KEY(Periodontitis) OR TITLE-ABS-KEY(Periodontitides) OR TITLE-ABS-KEY(Pericementitis) OR TITLE-ABS-KEY(Pericementitis) OR TITLE-ABS-KEY("Periodontal Diseases") OR TITLE-ABS-KEY("Disease, Periodontal") OR TITLE-ABS-KEY("Diseases, Periodontal") OR TITLE-ABS-KEY("Periodontal Disease") OR TITLE-ABS-KEY(Parodontosis) OR TITLE-ABS-KEY(Parodontoses) OR TITLE-ABS-KEY("Pyorrhea Alveolaris") OR TITLE-ABS-KEY("Periodontal Atrophy") OR TITLE-ABS-KEY("Periodontal Atrophies") OR TITLE-ABS-KEY("Atrophy of Periodontium") OR TITLE-ABS-KEY("Periodontium Atrophies") OR TITLE-ABS-KEY("Periodontium Atrophy") OR TITLE-ABS-KEY("Gingivo-Osseous Atrophy") OR TITLE-ABS-KEY("Gingivo Osseous Atrophy") OR TITLE-ABS-KEY("Gingivo-Osseous Atrophies") OR TITLE-ABS-KEY("Alveolar Bone Loss") OR TITLE-ABS-KEY("Alveolar Bone Losses") OR TITLE-ABS-KEY("Alveolar Process Atrophy") OR TITLE-ABS-KEY("Alveolar Process Atrophies") OR TITLE-ABS-KEY("Alveolar Resorption") OR TITLE-ABS-KEY("Alveolar Resorptions") OR TITLE-ABS-KEY("Resorption, Alveolar") OR TITLE-ABS-KEY("Resorptions, Alveolar") OR TITLE-ABS-KEY("Bone Loss, Periodontal") OR TITLE-ABS-KEY("Bone Losses, Periodontal") OR TITLE-ABS-KEY("Periodontal Bone Losses") OR TITLE-ABS-KEY("Periodontal Bone Loss") OR TITLE-ABS-KEY("Periodontal Resorption") OR TITLE-ABS-KEY("Periodontal Resorptions") OR TITLE-ABS-KEY("Resorption, Periodontal") OR TITLE-ABS-KEY("Alveolar Bone Atrophy") OR TITLE-ABS-KEY("Alveolar Bone Atrophies") OR TITLE-ABS-KEY("Bone Atrophies, Alveolar") OR TITLE-ABS-KEY("Bone Atrophy, Alveolar") OR TITLE-ABS-KEY("Bone Loss, Alveolar") OR TITLE-ABS-KEY("Chronic Periodontitis") OR TITLE-ABS-KEY("Chronic Periodontitides") OR TITLE-ABS-KEY("Periodontitides, Chronic") OR TITLE-ABS-KEY("Periodontitis, Chronic") OR TITLE-ABS-KEY("Adult Periodontitis") OR TITLE-ABS-KEY("Adult Periodontitides") OR TITLE-ABS-KEY("Periodontitides, Adult") OR TITLE-ABS-KEY("Periodontitis, Adult"))) AND ((TITLE-ABS-KEY(Humans) OR TITLE-ABS-KEY("Man,Modern") OR TITLE-ABS-KEY("Modern Man") OR TITLE-ABS-KEY("Man Taxonomy") OR TITLE-ABS-KEY("Homo sapiens") OR TITLE-ABS-KEY("Human") OR TITLE-ABS-KEY(Adults) OR TITLE-ABS-KEY(Adult))) AND ((TITLE-ABS-KEY(Hydrocortisone) OR TITLE-ABS-KEY("Cortisol,dehydroepiandrosterone") OR TITLE-ABS-KEY(Cortisol) OR TITLE-ABS-KEY("Hydrocortisone, (9 beta,10 alpha,11 alpha)-Isomer") OR TITLE-ABS-KEY("Hydrocortisone,(11 alpha)-Isomer") OR TITLE-ABS-KEY("11-Epicortisol") OR TITLE-ABS-KEY("11 Epicortisol") OR TITLE-ABS-KEY(Epicortisol) OR TITLE-ABS-KEY(Cortifair) OR TITLE-ABS-KEY(Cortril) OR TITLE-ABS-KEY("Stress,Psychological") OR TITLE-ABS-KEY("Psychological Stresses") OR TITLE-ABS-KEY("Stresses,Psychological") OR TITLE-ABS-KEY("Life Stress") OR TITLE-ABS-KEY("Life Stresses") OR TITLE-ABS-KEY("Stress,Life") OR TITLE-ABS-KEY("Stresses,Life") OR TITLE-ABS-KEY("Stress,Psychologic") OR TITLE-ABS-KEY("Psychologic Stress") OR TITLE-ABS-KEY("-Psychological Stress") OR TITLE-ABS-KEY("Mental Suffering") OR TITLE-ABS-KEY("Suffering,Mental") OR TITLE-ABS-KEY(Suffering) OR TITLE-ABS-KEY(Anguish) OR TITLE-ABS-KEY("Emotional Stress") OR TITLE-ABS-KEY("Stress,Emotional")))
COCHRANE	Human$ or Man, Modern or "Modern man" or "Man (taxonomy)" or "Homo sapiens" or "Human" or Adult or Adults and Hydrocortisone or "Cortisol,dehydroepiandrosterone" or Cortisol or "Hydrocortisone, (9 beta,10 alpha,11 alpha)-Isomer" or "Hydrocortisone, (11 alpha)-Isomer" or "11-Epicortisol" or "11 Epicortisol" or Epicortisol or Cortifair or Cortril or Stress, Psychological or "Psychological Stresses" or Stresses, Psychological or "Life Stress$" or Stress, Life or Stresses, Life or Stress, Psychologic or "Psychologic Stress" or "Psychological Stress" or "Mental Suffering" or Suffering, Mental or Suffering or Anguish or "Emotional Stress" or Stress, Emotional AND periodontitis or Periodontitides or "Chronic Periodontitis" or "Agressive Periodontitis" or "Periapical periodontitis" or "Periodontal desease$" Disease, Periodontal or Diseases, Periodontal or Parodontosis or Parodontoses or "Pyorrhea Alveolaris" or "Alveolar Bone Loss$" or "Alveolar Process Atroph$" or "Alveolar Resorption$" or Resorption, Alveolar or Resorptions, Alveolar or Bone Loss, Periodontal or Bone Losses, Periodontal or "Periodontal Bone Loss$" or "Periodontal Resorption$" or Resorption, Periodontal or "Alveolar Bone Atroph$" or "Bone Atroph$, Alveolar" or "Bone Loss, Alveolar" or "Periodontal Atroph$" or "Atrophy of Periodontium" or "Periodontium Atroph$" or "Gingivo-Osseous Atroph$" or "Gingivo Osseous Atrophy" AND "Chronic Periodontitis" or "Chronic Periodontitides" or Periodontitides, Chronic or Periodontitis, Chronic or "Adult Periodontitis" or "Adult Periodontitides" or Periodontitides, Adult or Periodontitis, Adult

(*continued*)

Supplementary Table S2 (*continued*)

Database	Search Format
WEB OF SCIENCE	TS=(Humans OR “Man, Modern” OR “Modern Man” OR “Man Taxonomy” OR “Homo sapien” OR Human OR adult OR adults AND Hydrocortisone OR “Cortisol,dehydroepiandrosterone” OR “Cortisol” OR “Hydrocortisone, (9 beta,10 alpha,11 alpha)-Isomer” OR “Hydrocortisone, (11 alpha)-Isomer” OR “11-Epicortisol” OR “11 Epicortisol” OR Epicortisol OR Cortifair OR Cortril OR “Stress, Psychological” OR “Psychological Stresses” OR “Stresses, Psychological” OR “Life Stress” OR “Life Stresses” OR “Stress, Life” OR “Stresses, Life” OR “Stress, Psychologic” OR “Psychologic Stress” OR “Psychological Stress” OR “Mental Suffering” OR “Suffering, Mental” OR Suffering OR Anguish OR “Emotional Stress” OR “Stress, Emotional” AND Periodontitis OR Periodontitides OR Pericementitis OR Pericementitides OR “Periodontal Diseases” OR “Disease, Periodontal” OR “Diseases, Periodontal” OR “Periodontal Disease” OR Parodontosis OR Parodontoses OR “Pyorrhea Alveolaris” OR “Periodontal Atrophy” OR “Periodontal Atrophies” OR “Atrophy of Periodontium” OR “Periodontium Atrophies” OR “Periodontium Atrophy” OR “Gingivo-Osseous Atrophy” OR “Gingivo Osseous Atrophy” OR “Gingivo-Osseous Atrophies” OR “Alveolar Bone Loss” OR “Alveolar Bone Losses” OR “Alveolar Process Atrophy” OR “Alveolar Process Atrophies” OR “Alveolar Resorption” OR “Alveolar Resorptions” OR “Resorption, Alveolar” OR “Resorptions, Alveolar” OR “Bone Loss, Periodontal” OR “Bone Losses, Periodontal” OR “Periodontal Bone Losses” OR “Periodontal Bone Loss” OR “Periodontal Resorption” OR “Periodontal Resorptions” OR “Resorption, Periodontal” OR “Alveolar Bone Atrophy” OR “Alveolar Bone Atrophies” OR “Bone Atrophies, Alveolar” OR “Bone Atrophy, Alveolar” OR “Bone Loss, Alveolar” OR “Chronic Periodontitis” OR “Chronic Periodontitides” OR “Periodontitides, Chronic” OR “Periodontitis, Chronic” OR “Adult Periodontitis” OR “Adult Periodontitides” OR “Periodontitides, Adult” OR “Periodontitis, Adult”)
OPENGREY	Stress AND Periodontitis
LILACS	(Human$) OR (Man, Modern) OR (Modern Man) OR (Man Taxonomy) OR (Homo sapiens) OR (Human) OR (Adult) OR (Adults) AND (Hydrocortisone) OR (Cortisol,dehydroepiandrosterone) OR (Cortisol) OR (Hydrocortisone, (9 beta,10 alpha,11 alpha)-Isomer) OR (Hydrocortisone, (11 alpha)-Isomer) OR (11-Epicortisol) OR (11 Epicortisol) OR (Epicortisol) OR (Cortifair) OR (Cortril) OR (Stress$, Psychological) OR (Psychological Stresses) OR (Cortisol) OR (Life Stress$) OR (Stress$, Life) OR (Stress, Psychologic) OR (Psychologic Stress$) OR (Mental Suffering) OR (Suffering, Mental) OR (Suffering) OR (Anguish) OR (Emotional Stress) OR (Stress, Emotional) AND (Chronic Periodontitis) OR (Chronic Periodontitides) OR (Periodontitides, Chronic) OR (Periodontitis, Chronic) OR (Adult Periodontitis) OR (Adult Periodontitides) OR (Periodontitides, Adult) OR (Periodontitis, Adult) OR (periodontitis) OR (Periodontitides) OR (Chronic periodontitis) OR (Aggressive Periodontitis) OR (Periapical Periodontitis) OR (Periodontal Disease$) OR (Disease$, Periodontal) OR (Parodontosis) OR (Parodontoses) OR (Pyorrhea Alveolaris) OR (Periodontal Atroph$) OR (Atrophy of Periodontium) OR (Periodontium Atroph$) OR (Gingivo-Osseous Atrophy) OR (Gingivo Osseous Atroph$) OR (Alveolar Bone Loss$) OR (Alveolar Process Atroph$) OR (Alveolar Resorption$) OR (Resorption$, Alveolar) OR (Bone Loss$, Periodontal) OR (Periodontal Bone Loss$) OR (Periodontal Resorption$) OR (Resorption, Periodontal) OR (Alveolar Bone Atroph$) OR (Bone Atroph$, Alveolar) OR (Bone Loss, Alveolar)
GOOGLE SCHOLAR	Humans+Periodontitis+Stress-Review
Clinical trials	Periodontitis AND Stress
